# Bone-Derived Factors as Potential Biomarkers for Parkinson’s Disease

**DOI:** 10.3389/fnagi.2021.634213

**Published:** 2021-02-24

**Authors:** Yuwan Lin, Miaomiao Zhou, Wei Dai, Wenyuan Guo, Jiewen Qiu, Zhiling Zhang, Mingshu Mo, Liuyan Ding, Panghai Ye, Yijuan Wu, Xiaoqin Zhu, Zhuohua Wu, Pingyi Xu, Xiang Chen

**Affiliations:** ^1^Department of Neurology, The First Affiliated Hospital of Guangzhou Medical University, Guangzhou, China; ^2^Department of Physiology, School of Basic Medical Sciences, Guangzhou Medical University, Guangzhou, China

**Keywords:** Parkinson’s disease, bone-derived factors, osteocalcin, osteopontin, C-reaction protein

## Abstract

**Background**: Parkinson’s disease (PD) and osteoporosis are both common aging diseases. It is reported that PD has a close relationship with osteoporosis and bone secretory proteins may be involved in disease progression.

**Objectives**: To detect the bone-derived factors in plasma and cerebrospinal fluid (CSF) of patients with PD and evaluate their correlations with C-reaction protein (CRP) level, motor impairment, and Hoehn-Yahr (HY) stage of the disease.

**Methods**: We included 250 PD patients and 250 controls. Levels of osteocalcin (OCN), osteopontin (OPN), osteoprotegerin (OPG), Sclerostin (SO), Bone morphogenetic protein 2 (BMP2), and Dickkopf-1 (DKK-1) in plasma and CSF were measured by custom protein antibody arrays. Data were analyzed using Mann–Whitney *U*-test and Spearman’s receptor activator of NF-κB (RANK) correlation.

**Results**: Plasma levels of OCN and OPN were correlated with CRP levels and HY stage and motor impairment of PD. Furthermore, the plasma assessment with CSF detection may enhance their potential prediction on PD.

**Conclusions**: OCN and OPN may serve as potential biomarkers for PD. The inflammation response may be involved in the cross-talk between the two factors and PD.

## Introduction

Parkinson’s disease (PD) is one of the most common neurodegenerative disorders. It is investigated that approximately 7.4–23.4% of the patients with PD suffered from osteoporosis as a result of immobility, vitamin D deficiency, hyperhomocysteinemia, malnutrition, and muscle weakness (Choi et al., 2017). Besides, fractures can also be found more commonly in the prodromal period of PD compared to controls (Camacho-Soto et al., 2020). Both of these indicate that osteoporosis may be a hidden non-motor syndrome of PD and bone metabolism has a close relationship with the development of PD (Metta et al., 2017). Bone has traditionally been considered a structural organ that supports the movement of the body and protects the internal organs. However, an increasing number of studies have shown that the skeleton could also be an active endocrine organ that secretes many kinds of bone-derived factors and contributes to the pathophysiology of many diseases, such as Alzheimer’s disease, diabetes mellitus, cardiovascular, chronic kidney disease (Rentsendorj et al., 2018; Xu et al., 2018). In this study, we aimed to clarify the levels of OCN, OPN, OPG, SO, BMP2, and DKK-1 in the plasma and CSF of PD patients and identify candidate biomarkers for detection of PD. We also investigated the relevance of these bone-derived factors with CRP and motor dysfunction and disease progression.

## Materials and Methods

### Subjects

This study enrolled 500 participants, including 250 PD, and 250 healthy controls (HCs). Among the participants, 400 were collected for plasma (200 PD, 200 HCs), 100 were collected for CSF (50 PD, 50 controls). PD patients were diagnosed following the Movement Disorder Society Clinical Diagnostic Criteria. All patients included in this study were tested negative for secondary forms of parkinsonism, neurological/psychiatric, diabetes mellitus, thyroid disease, history of metabolic bone disorders, renal dysfunction and failure, and severe cardiovascular and systemic diseases affecting the overall health of participants. Controls were matched with PD patients regarding their age and gender. All participants were recruited from the First Affiliated Hospital of Guangzhou Medical University. This study was approved by the Institutional Ethics Board Committee of the First Affiliated Hospital of Guangzhou Medical University and all participants provided written informed consent.

### Plasma and CSF Collection

The blood samples of 200 HCs and 200 patients with PD were collected in early morning (7 a.m. – 9 a.m. ) and were centrifuged (2,500 *g* for 15 min at 4° C) within 1 h of the collection (Redmond et al., 2016). CSF samples were collected by lumbar puncture from 50 controls and 50 patients with PD. CSF samples had no blood contamination (leukocyte number count fewer than 5 cells/μl and erythrocyte number fewer than 200 cells/μl). Sample aliquots were stored in cryotubes at −80°C before testing.

### Measurement of Bone-Derived Factors in Plasma and CSF

The levels of bone-derived factors were measured using custom protein antibody arrays (RayBiotech^1^). First of all, a captured antibody was bound to the glass surface. Then the standard protein cocktail, 50–100 μl plasma or CSF samples were added to the platform and incubated for 1–2 h. Second, a second biotin-labeled detection antibody was added and incubated for 1–2 h, which can recognize a different epitope of bone-derived factors. Next, the streptavidin-conjugated Cy3 equivalent dye which could visualize the cytokine-antibody-biotin complex was added and incubated for 1 h. Last, the data was scanned and performed by using a laser scanner.

### Measurement of CRP in Plasma and CSF

The levels of CRP in plasma and CSF were measured by immunoturbidimetric assays. For the estimation of CRP levels, an automatic biochemical analyzer was used according to the manual instruction.

### Statistical Analysis

Data statistics were carried out by SPSS, version 21, and GraphPad Prism version 6. Student’s *t*-tests or Mann–Whitney *U* test was used to assess the difference of continuous variables between PD and HC groups. Differences between groups for categorical variables were assessed by using chi-square tests. Spearman coefficient calculation and the Kruskal–Wallis *H* test were used to analyze possible correlations between parameters of interest. Receiver operating characteristics (ROC) curves were used to determine the diagnostic performance of studied bone-derived factors in differentiating PD patients from controls. The accuracy of a biomarker in predicting PD was assessed by calculating the area under the ROC curve (AUC). Values of *P* < 0.05 were considered significant.

## Results

**Figure 1:**
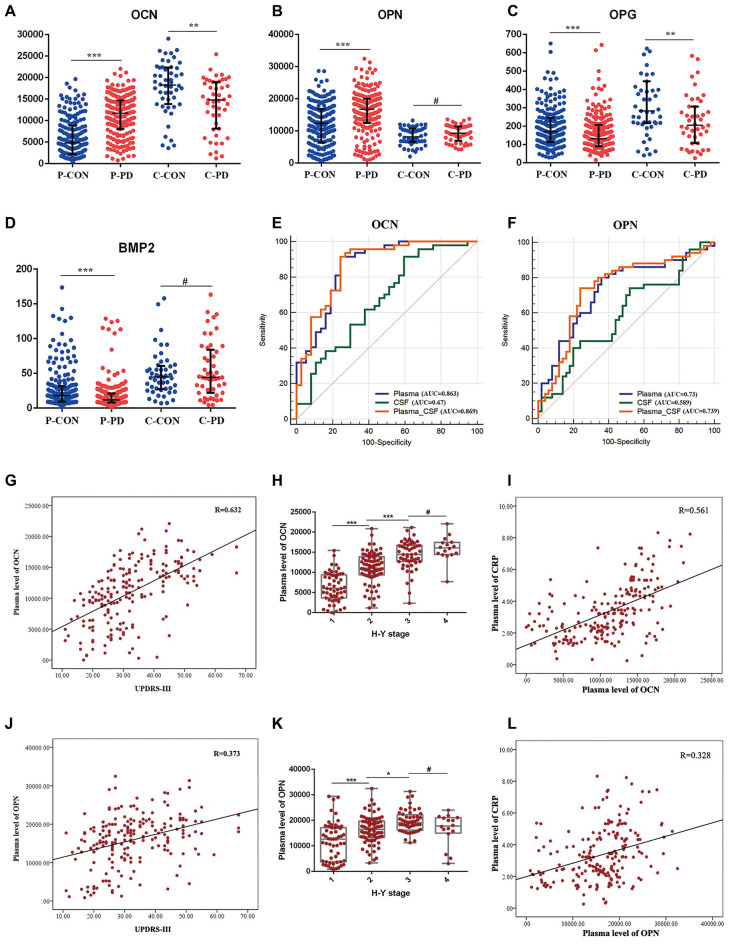
Expression levels, receiver operating characteristics (ROC) analysis, and correlation analysis of selected bone-derived factors of Parkinson’s disease (PD) relative to healthy control (HC). **(A–D)** Presents the concentrations of osteocalcin (OCN), osteopontin (OPN), osteoprotegerin (OPG), and bone morphogenetic protein 2 (BMP2) 1 in plasma (P) and cerebrospinal fluid (CSF; **C**) of PD and HC (CON). Data are presented as median and interquartile range (IQR; **P < 0.01, ***P < 0.001, ^#^*P* = 0.05 from Mann–Whitney test). **(E,F)** ROC curves of plasma, CSF, and combined plasma and CSF (Plasma-CSF) of OCN and OPN were analyzed. AUC, area under the curve. **(G)** Scatter diagram of the correlation between the plasma level of OCN and Unified Parkinson’s Disease Rating Scale part III (UPDRS-III) of PD patients (*r* = 0.632, *P* < 0.001). **(H)** The relationship of plasma level of OCN and Hoehn and Yahr (H-Y) stage of PD patients, (***P < 0.001, *P < 0.05, ^#^*P* = 0.05). **(I)** Correlation between the plasma level of OCN and C-reaction protein (CRP) of PD patients (*r* = 0.561, *P* < 0.001). **(J)** Scatter diagram of the correlation between the plasma level of OPN and UPDRS-III of PD patients (*r* = 0.373, *P* < 0.001). **(K)** The relationship of plasma level of OPN and H-Y stage of PD patients (***P < 0.001 *P < 0.05, ^#^*P* = 0.05). **(L)** Correlation between the plasma level of OPN and CRP of PD patients (*r* = 0.328, *P* < 0.001).

For this study, we obtained plasma samples from 200 PD patients and 200 age- and gender-matched controls, and CSF samples from 50 PD patients and 50 age- and gender-matched controls. The detectable rates of six bone-derived factors in plasma and five bone-derived factors in CSF were more than 90%. SO in CSF analysis was excluded due to the low detection rate (52%). In plasma, levels of OCN [PD vs. HC, 11,716.4pg/ml (8,052.7–14,679.4) vs. 4833.1 pg/ml (1,953.1–8,847.8), *P* < 0.001] and OPN [PD vs. HC, 16,733.7 pg/ml (12,446.0–19,981.3)vs 12,333.7 pg/ml (6,341.4–16,882.7), *P* < 0.001] were increased in the PD patients relative to the controls. In contrast, levels of OPG [PD vs HC, 130.7 pg/ml (89.4–207) vs. 169.5 pg/ml (113.1–245), *P* < 0.001] and BMP2 [PD vs. HC, 11.8 pg/ml (8–20.9) vs. 17.9 pg/ml (9.37–31.5), *P* < 0.001] were decreased in the PD patients compared to those in controls (Table 1, Figures 1A–D). However, no significant difference in plasma SO and DKK1 levels were found between the two groups (Supplementary Figure 1). In CSF, patients with PD had significantly lower levels of OCN [14,817.5 pg/ml (8,145.9–18,998.3) vs. 18,264 pg/ml (12,835.5–22,342.3), *P* = 0.002; Figure 1A] and OPG [204.3 pg/ml (107.3–307) vs. 282.7 pg/ml (215.3–444.1), *P* = 0.008; Figure 1C] relative to HC. There was no significant difference in OPN, BMP2, or DKK1 levels in CSF between the two groups (Table 1, Figures 1B,D, and Supplementary Figure 1).

**Table 1:** Demographics and protein levels of six bone-derived factors in plasma and cerebrospinal fluid (CSF) in two groups.

Clinical characteristics	Plasma		*P*-value	CSF		*P*-value
	PD	Con		PD	Con	
Gender (Male/Female)	113/87	108/92	0.688	29/21	27/23	0.84
Age (SD), years	63.2 (11.2)	63.3 (12.3)	0.935	57.6 (11.1)	59 (10.2)	0.518
SBP (SD), mm Hg	122.2 (10.32)	123.6 (11.9)	0.298	121.4 (12.5)	122.4 (10.6)	0.579
DBP (SD), mm Hg	75.2 (7.0)	74.6 (7.5)	0.215	74.5 (7.3)	73.9 (7.5)	0.553
H-Y	2.3 (0.8)	-	-	2.2 (0.6)	-	-
UPDRS-III	33.1 (11.6)	-	-	32.4 (11.7)	-	-
CRP, median (IQR), mg/l	3.3 (1.8–4.5)	2.1 (1.1–2.9)	<0.001	0.15 (0.12–0.17)	0.032 (0.01–0.04)	0.006
OCN, median (IQR), pg/ml	11,716.4 (8052.7–14,679.4)	4,833.1 (1953.1–8,847.8)	<0.001	14,817.5 (8145.9–18,998.3)	18,264 (12835.5–22,342.3)	0.002
OPN, median (IQR), pg/ml	16,733.7 (12446.0–19,981.3)	12,333.7 (6341.4–16,882.7)	<0.001	93,16.1 (6955.1–11,378.3)	8,189.4 (6629.2–10736.8)	0.124
OPG, median (IQR), pg/ml	130.7 (89.4–207)	169.5 (113.1–245)	<0.001	204.3 (107.3–307)	282.7 (215.3–444.1)	0.008
SO, median (IQR), pg/ml	651.4 (321.1–1068.7)	748.6 (372.6–1272.2)	0.147	-	-	-
BMP2, median (IQR), pg/ml	11.8 (8–20.9)	17.9 (9.37–31.5)	<0.001	44.3 (22–84.1)	45 (27.5–60.5)	0.900
DKK1, median (IQR), pg/ml	45.6 (15.8–141.1)	63.6 (24.8–135.4)	0.067	16.2 (5.2–56.3)	33.46 (18.1–54.7)	0.080

*Data are represented as Mean (SD), median (IQR), or *n*. *P*-value was considered significant *when* < 0.05, PD, Parkinson’s disease; con, Controls; SBP, systolic blood pressure; DBP, diastolic blood pressure; H-Y, Hoehn and Yahr, UPDRS-III, Unified Parkinson’s Disease Rating Scale part III (motor score). CRP, C-reaction protein; OCN, Osteocalcin; OPN, Osteopontin; OPG, Osteoprotegerin; SO, Sclerostin; BMP2, Bone morphogenetic protein 2; DKK-1, Dickkopf-1; IQR, Interquartile range*.

To evaluate whether these bone-derived factors could be the potential biomarkers for PD risk, the natural-logarithm values of these levels were analyzed using ROC curves. Compared with the HC group, the AUCs for the plasma and CSF levels of OCN in PD patients were 0.863 (95% *CI* = 0.771–0.928) and 0.67 (95% *CI* = 0.559–0.769), respectively. Moreover, the AUC was higher when the combined assessment of plasma and CSF OCN in PD patients at 0.869 (95% *CI* = 0.778–0.993; Figure 1E). The AUCs for plasma, CSF, and the combined assessment of OPN when comparing PD patients with control groups were 0.73 (95% *CI* = 0.632–0.814), 0.589 (95% *CI* = 0.486–0.687), and 0.739 (95% *CI* = 0.641–0.822), respectively (Figure 1F). The AUCs for plasma, CSF, and the combined assessment of OPG and BMP2 were less than 0.7 (Supplementary Figure 1). Further, the plasma level of OCN and OPN were correlated with the Hoehn and Yahr disease stage (Figures 1H–K) and the Movement Disorders Society-Unified Parkinson’s Disease Rating Scale-III score (MDS-UPDRS III; Figures 1G–J). Inflammation was involved both in the pathology of osteoporosis and PD, we next analyzed the relationship between CRP and these bone-derived factors. We found that significant correlations existed between plasma OCN and OPN and CRP levels in PD (Figures 1I–L). However, no correlation was identified between these factors and CRP in CSF.

## Discussion

Several studies have focused on the questions of comorbidity of PD and osteoporosis. The relationship between bone-derived factors and the risk of PD remains unclear. In the present study, we tested six bone-derived factors and found increased levels of OCN and OPN and decreased levels of OPG and BMP2 in plasma of PD patients. And levels of OCN and OPG were lower in CSF of PD relative to controls. Furthermore, we identified that plasma OCN and OPN were correlated with the disease stage and motor impairment. CRP was correlated with plasma levels of OCN and OPN. Combined assessment of plasma and CSF of OCN or OPN would be a better biomarker for differentiating PD patients from HCs.

OCN is one of the most abundant bone-specific non-collagenous proteins secreted primarily by osteoblasts and is often used as a biomarker for bone formation. In recent years, OCN has been regarded as a bone-derived hormone that plays important roles in physiological and pathological processes (Wei and Karsenty, 2015). In the brain, the uncarboxylated form of OCN can accumulate in the brainstem, thalamus, and hypothalamus, influencing various neurotransmitters synthesis and signalings (Shan et al., 2019). Several types of research indicated that OCN offers its protective function in PD. OCN can bind with the neurons in the midbrain and interacts with the dopamine transporter through heterotrimeric G protein’s βγ subunit, and thus facilitating the formation of dopamine neurotransmitters (Garcia-Olivares et al., 2017). Intervention with OCN could relieve the behavioral dysfunction symptoms and reduce the tyrosine hydroxylase loss in the nigrostriatal system in PD rat models (Guo et al., 2018). Also, OCN could modulate neuroinflammation in the substantia nigra (SN) of PD rats by inhibiting astrocyte and microglia proliferation, together with partially decreased levels of TNF-α and IL-1β. Further studies confirmed that OCN corrected motor dysfunction, inhibited the neuroinflammatory responses, and reduced dopaminergic neuronal injury *via* the AKT/GSK3β signaling pathway in an animal model of PD (Guo et al., 2018). Additionally, OCN has an important role in brain development. OCN is required during embryonic development for proper neuronal development (Moser and van der Eerden, 2018). In OCN-deficient mice, the brains were smaller and less developed than control mice. OCN was also indicated to have a significant impact on cognition. Serum OCN levels were correlated with cognitive performance in aged women and obese patients (Bradburn et al., 2016; Puig et al., 2016). OCN-deficient mice were more susceptible to suffer from cognitive defects and exogenous OCN could protect against cognitive function in mice (Khrimian et al., 2017). In the peripheral, OCN acts as a regulator of the activity of osteoclasts and also maintains the energy homeostasis by improving glucose metabolism, insulin sensitivity (Mizokami et al., 2017). OCN also plays an important role in inflammation. The OCN treatment in obese mice could reduce substantially the expression of proinflammatory cytokines, chemokine, and inflammasome-related genes. Furthermore, OCN treatment prevents infiltration of lymphocytes and fibrosis and reduces the density of macrophages in crown-like structures (Guedes et al., 2018). Also, both carboxylated and uncarboxylated OCN increase secretion of adiponectin and the anti-inflammatory cytokine interleukin 10 (Hill et al., 2014). In this study, we found that the plasma level of OCN was increased, however, the CSF level of OCN was decreased in PD patients. This may be accounted for by the different roles of OCN in peripheral circulation and the central nervous system. Given the role of OCN in protecting dopaminergic neurons, improving cognition, and preventing anxiety and depression the low expression level of OCN in CSF indicates its involvement in motor and non-motor symptoms of PD. Plasma level of OCN correlated with CRP and H-Y stage of PD patients indicates inflammation may be a potential bridge between OCN and progression of PD. However, no correlation was identified between OCN and CRP in CSF, indicating that other mechanisms are involved in PD in the central nervous system. Furthermore, compared to analyzing plasma or CSF OCN alone, the combined assessment is more effective in differentiating PD patients from HCs, and the plasma level of OCN was correlated with the disease stage and motor impairment. All these results highlight the essential roles of OCN in the pathology of PD.

OPN is a glycosylated phosphoprotein belonging to the small integrin-binding ligand, N-linked glycoprotein (SIBLING) family of proteins (Pang et al., 2019). It is highly expressed by bone marrow-derived myelomonocytic cells and can act both as a matrix protein and as a cytokine. As a multifunctional protein, OPN plays significant roles in regulating reactive oxygen species production, levels of inflammatory cytokines, and apoptotic signals (Rentsendorj et al., 2018). OPN was reported to be involved in the pathology of various brain diseases, such as Alzheimer’s disease (AD), multiple sclerosis, and traumatic brain injury *via* neuroprotective and repair-promoting effects (Carecchio and Comi, 2011; Rentsendorj et al., 2018). In PD, the function of OPN mainly derives from its anti-inflammatory and anti-apoptotic properties, as well as its role in regulating reactive oxygen species (ROS) production, and cytokine levels (Khan et al., 2002; Lund et al., 2009; Rittling and Singh, 2015). Also, OPN is expressed in SN and in nigral dopaminergic neurons (DAs) and its expression is decreased in surviving dopaminergic neurons in PD, suggesting a potential role of OPN in neuroprotection of PD. Further study showed that the arginine-glycine-aspartic acid (RGD)-containing domain of OPN, could protect tyrosine hydroxylase (TH)-positive cells against toxic insult induced by MPP^+^ and LPS, indicating that this peptide fragment of OPN may be necessary for the survival of TH cells and have neuroprotective properties relevant to Parkinson’s disease (Iczkiewicz et al., 2010). However, OPN knockout mice displayed less nigral cell death and a decreased glial response in a 1-methyl-4-phenyl-1,2,3,6-tetrahydropyridine (MPTP) induced animal model of PD (Maetzler et al., 2007; Carecchio and Comi, 2011). This suggests that OPN may act as a double-edged sword triggering neuronal toxicity or functioning as a neuroprotectant in PD. The inflammatory response may be one of the mechanisms of OPN in PD as there is a significant correlation between OPN and CRP. Maetzler et al. (2007) have reported that OPN was upregulated both in the plasma and CSF of PD patients, however, we only identified a higher level of OCN in the plasma of PD patients. The incongruence might be owing to the difference in disease stage, ethnic or environmental factors and more studies are needed to reveal the function of OPN in PD.

OPG is a tumor necrosis factor (TNF) receptor superfamily protein and expressed mainly by bone marrow stromal cells, but can also be induced in B lymphocytes and dendritic cells (Schoppet et al., 2007; Li et al., 2014). OPG plays essential roles in receptor activator of nuclear factor kappa-B (NF-kB) ligand (RANKL)–receptor activator of NF-κB (RANK)–OPG axis to regulate bone metabolisms, organogenesis, immune tolerance, and cancer (Walsh and Choi, 2014). In this study, although OPG may not be a biomarker for PD, the decreased levels of OPG in the plasma and CSF of PD patients indicated its involvement in the pathophysiology of the disease. In a small sample size study (*N* = 26), the serum levels of OPG were higher in PD patients than in controls (*P* = 0.04); (Alrafiah et al., 2019). However, in an animal model of AD, the plasma levels of OPG were down-regulated (Ali et al., 2019). The different results may be due to the sample size, disease severity, or detection sensitivity, and further investigations with a larger sample size are required to verify the findings of the present study.

BMP2 is a transforming growth factor-β (TGF-β) superfamily protein that plays significant roles in skeletal development, anti-inflammatory, and embryonic development (Grgurevic et al., 2016). In PD, BMP2 can facilitate the transformation of neural stem cells to DAs and promote neurite growth (Jordan et al., 1997). BMP2 overexpression can protect against neurotoxin-induced or A53T-α-synuclein-induced DAs degeneration by canonical sma and mothers against decapentaplegic (Smad) signaling pathway (Goulding et al., 2019). In this study, we found that BMP2 was downregulated in the plasma of PD patients than in HCs. All these results indicate that BMP2 may act as a neurotrophic factor for PD.

In conclusion, we analyzed six bone-derived factors and revealed abnormal expression levels of OCN, OPN, OPG, and BMP2 in plasma or CSF of PD. We identified that plasma levels of OCN and OPN were correlated with CRP, H-Y stage, and motor impairment. Our study also suggests that combined assessment of plasma and CSF of OCN may enhance their potential for predicting PD. Inflammation response may be involved in the cross-talk between these two factors and PD. These findings may contribute to the functional understanding of PD pathophysiology. However, further studies are needed to confirm our findings and to illustrate the roles of inflammation or immune mechanisms involved in the factors on PD.

## Data Availability Statement

The originale contributions presented in the study are included in the article, further inquiries can be directed to the corresponding authors.

## Ethics Statement

The studies involving human participants were reviewed and approved by Medical Ethics Committee of The First Affiliated Hospital of Guangzhou Medical University. The patients/participants provided their written informed consent to participate in this study.

## Author Contributions

PX and ZW: research project conception. XC and MM: research project organization. YL, MZ, WD, JQ, LD, and PY: research project execution. WG, YW, ZZ, and MM: statistical analysis execution. ZW and XZ: statistical analysis—review and critique. XC: manuscript—writing of the first draft. ZW and PX: manuscript—review and critique. All authors read and approved the final manuscript.

## Conflict of Interest

The authors declare that the research was conducted in the absence of any commercial or financial relationships that could be construed as a potential conflict of interest.

## Footnotes

^1^http://raybiotech.com
