# Supplementary material for: Bone-Derived Factors as Potential Biomarkers for Parkinson’s Disease
Source: Front Aging Neurosci. 2021 Feb 24;13:634213. doi: 10.3389/fnagi.2021.634213 (PMC7959739; doi:10.3389/fnagi.2021.634213)
Supplement: Supplementary file 1 [file Image_1.pdf]

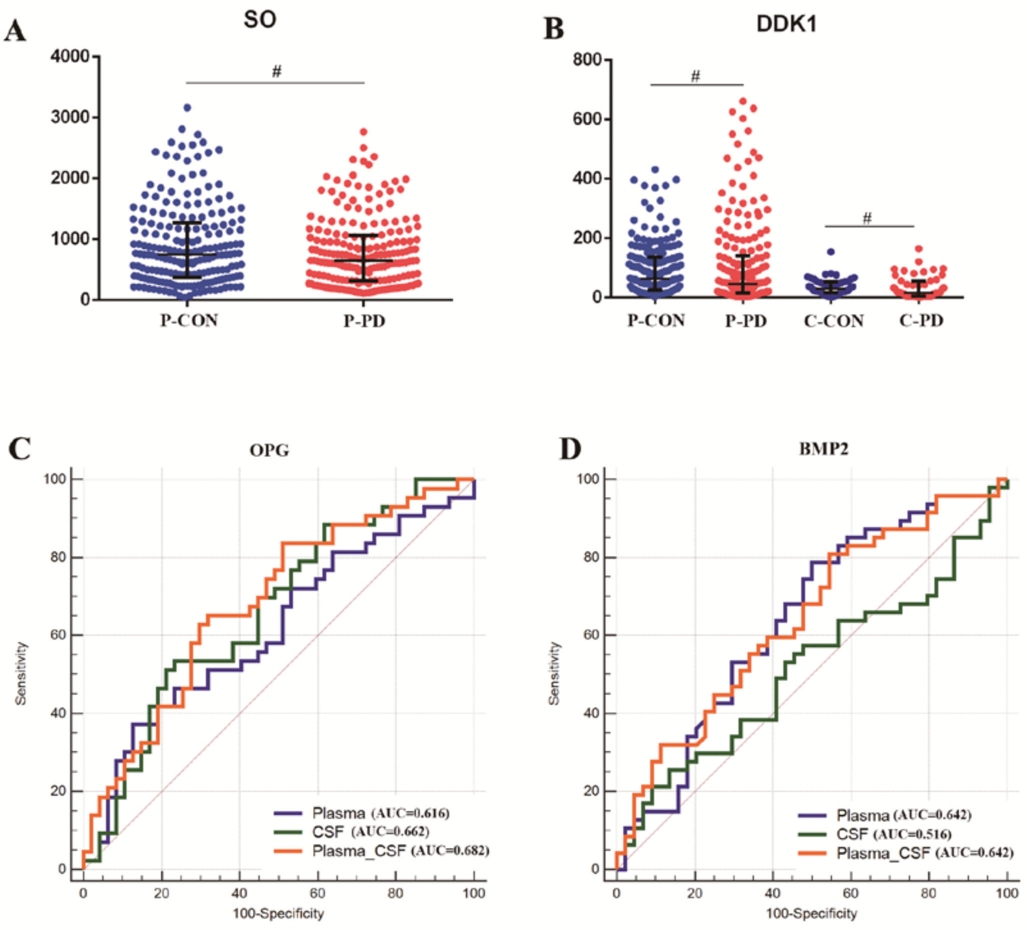

Figure1.A-B present the concentrations of SO and DKK1 in plasma (P) and cerebrospinal fluid (C) levels of Parkinson's disease (PD) and healthy controls (CON). Data are presented as median and IQR (**$P < 0.01$, ***$P < 0.001$, #$P > 0.05$). C-D, Receiver operating characteristics curves of plasma, CSF and combined of plasma and CSF (Plasma-CSF) of OPG and BMP2 were analyzed. AUC, area under the curve.
